# Corrosion Resistance of Boronized Carbon Steel in a Galinstan–Refrigerant Two-Phase Agitated System

**DOI:** 10.1021/acsomega.6c04193

**Published:** 2026-10-12

**Authors:** Lijun Wu, Eugene Medvedovski, Gerardo Leal Mendoza, Philip Geddis, Andrew McDonald

**Affiliations:** † Natural Resources Canada, CANMET Energy, 1 Haanel Dr, Ottawa, Ontario K1A 1M1, Canada; ‡ Advanced Materials Consultant, 39−C Stonehaven Dr, Ottawa, Ontario K2M 1E3, Canada; § 685727 Endurance Technologies Inc., #190, 550−71 Avenue SE, Calgary, Alberta T2H 0S6, Canada

## Abstract

This study experimentally evaluates, for the first time, the corrosion resistance of boronized carbon steel in environments containing liquid Galinstan (GaInSn) and selected organic refrigerants (*n*-pentane and R1233zd­(E)) at 150 °C for up to 80 days. The tests simulated gas–liquid-metal two-phase exposure under pseudodynamic conditions, where the test coupons and corrosive media were mechanically agitated. These conditions are relevant to advanced liquid-metal heat-transfer systems. Cross-sectional microstructural analysis revealed a uniform 180–200 μm thick FeB–Fe_2_B bilayer coating exhibiting a distinctive sawtooth morphology associated with strong adhesion to the substrate. Optical microscopy, hardness determination, and ICP-OES analysis indicated that the iron boride coating maintained its structural integrity after exposure. No evidence of surface degradation (e.g., cracking, caverns, peeling) or substrate degradation was observed by the employed characterization techniques. The coating thickness and microhardness showed no systematic decrease after exposure, remaining at their original levels. ICP-OES analysis revealed negligible concentrations of boron and iron (0.01–0.03 wt %) in the exposed Galinstan, with no systematic increase. Collectively, these observations indicated that no significant degradation or dissolution of the iron boride coating was detected during up to 80 days of exposure under the investigated conditions. In contrast, previously reported studies of SS316 identified measurable degradation under comparable conditions after only 20 days. These findings suggest that iron boride coatings warrant further investigation as a potentially cost-effective material option for selected liquid Galinstan-based heat-transfer systems. Additional studies involving larger sample populations, higher temperatures, longer exposure durations, and true dynamic flow conditions are required to further assess long-term performance and application potential.

## Graphical abstract

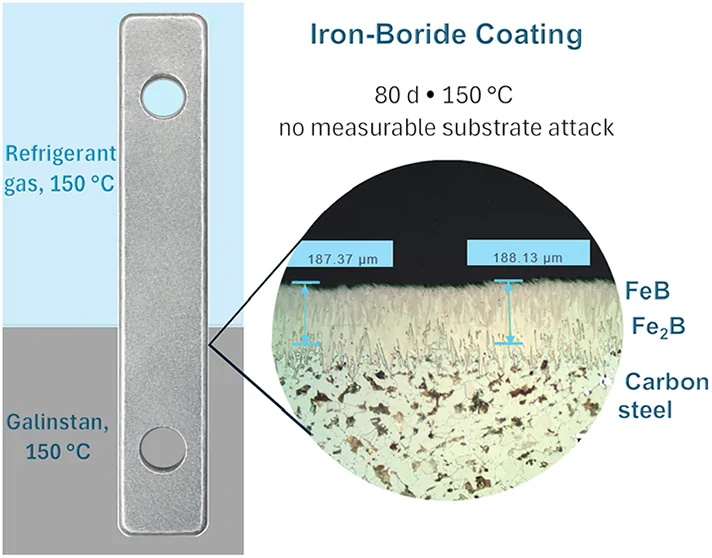

## 1 Introduction

Galinstan (GaInSn) is an alloy of eutectic Ga–In–Sn composition containing gallium (67 wt %), indium (20.5 wt %), and tin (12.5 wt %), with a low melting point of 10.5 °C. It remains liquid at room temperature and over a wide temperature range. Galinstan has a very low vapor pressure, which beneficially distinguishes it from the more hazardous mercury.^1,2^ Galinstan exhibits high thermal conductivity (∼16.5 W/m·K) and electrical conductivity (∼3.1 × 10^5^ S/m), both exceeding those of mercury at standard conditions. Its kinematic viscosity (∼3 × 10^–7^ m^2^/s) and density (∼6360 kg/m^3^) are lower than those of mercury, further supporting its advantageous fluid properties.^3^ The potential applications of Galinstan span both conventional and emerging technologies. Conventional applications include replacing mercury or other liquid metals for thermal regulation in nuclear reactors, such as use as a primary coolant.^4,5^ Emerging applications include the conversion of thermal or wave energy to electricity using liquid metals in magnetohydrodynamic (MHD) generators,^6^ as well as concentrated solar power, small modular reactors, nuclear fusion, electronics cooling – where compact and efficient heat transfer media are critically needed^7^ – and magnetic refrigeration.^8^ However, the corrosive nature of Galinstan, exacerbated by its low melting point and low viscosity, poses significant challenges for the integration of conventional solid metallic materials.

Corrosion of solid metallic materials in liquid-metal or molten metal environments commonly involves dissolution, intergranular penetration, and diffusion-assisted degradation. As with many corrosion processes, the material degradation starts from the surface defects, pitting, and gradual penetration of liquid metal. As a result, new corrosion products are formed on the surface and in the grain boundaries of the solid material.^9^ In the case of steels and alloys, new intermetallic compounds may be formed as corrosion products. These brittle intermetallic compounds with coefficients of thermal expansion (CTE), which differ from the CTE of the base material, peel off and create the surface and intergranular cracking.^10^ As an example, the exposure of stainless steels and Ni-based alloys in molten tin at 500 °C resulted in the depletion of Cr and Ni into liquid Sn with the formation of Ni–Sn intermetallics (e.g., Ni_3_Sn_4_).^11^ This may also lead to steel’s embrittlement.

The corrosion mechanisms described above are particularly relevant to Galinstan–steel systems. Gallium, the main element in Galinstan, has been found to be corrosive and was reported widely to cause the embrittlement of aluminum.^12^ It was found that gallium attacked 316L austenitic stainless steels and 1.4914 martensitic steel at a temperature of 400 °C.^13^ At 500 °C, SS316L experiences severe corrosion when exposed to liquid gallium and eutectic gallium alloys (Ga–Sn–Zn), and interaction leads to the formation of a thick, intermetallic reaction layer composed of matrix metals (Fe, Cr, Ni). Adding Sn and Zn to liquid gallium did reduce overall weight loss and reaction layer thickness compared to pure gallium, but insufficient to halt intermetallic layer formation and microfissuring, and protective surface coatings are critically required.^14,15^ A tensile test conducted at 300 °C showed that gallium did not embrittle type 316L stainless steel, but tests at room temperature showed that gallium did cause a slight decrease in ductility.^16^ At 200 °C exposed to Galinstan (Ga–In–Sn) under static conditions, nonferrous metals such as aluminum, copper, and brass showed severe corrosion after prolonged exposure, whereas ferritic and austenitic stainless steels and Ni–Cr-based alloys – including E26–1, SS304L, SS316, Inconel 725, and Hastelloy C2000 – exhibit significantly higher corrosion resistance. Polymeric materials, such as nitrile rubber, ABS, nylon, PVC, and Teflon, show satisfactory resistance only at lower temperatures (<100 °C).^17^

Besides the corrosive nature of liquid Gallium or Galinstan, its corrosive attack is defined by numerous factors. They include the presence of impurities, the nature and structure of the exposed solid material, the quality of the surface and interior microstructure (e.g., internal voids can accumulate liquid metal and corrosion products, facilitating degradation), material wettability, and process conditions such as temperature, liquid-metal flow velocity, gaseous environments, etc.

It is known that dynamic conditions (e.g., at agitation and, furthermore, at movement or flows of corrosive media) significantly accelerate corrosion, compared to static conditions, as the possibly occurring corrosion scale or protective film can be continuously removed, exposing fresh material to the corrosive stream. As a result, upon the scale or film removal, the degraded surface experiences facilitated degradation. When subjected to both the dynamic and elevated temperature conditions, which are typical of heat-transfer applications, material degradation can be further accelerated through hot liquid-metal flow.^5^ Additionally, in two-phase liquid-metal–gas flow conditions, such as those required for effective power generation through heat transfer in liquid-metal magnetohydrodynamic (LMMHD) systems, certain gaseous fluids or refrigerants are present together with liquid metals.^6,18,19^ The presence of a liquid–gas interface may create localized chemical and wetting conditions that differ from those encountered in single-phase conditions, and corrosion can potentially become even more severe due to the presence of gaseous chemicals. These considerations highlight the need for corrosion-resistant materials or protective surface treatments for components operating in Galinstan-containing two-phase systems.

SS316 has previously been evaluated for corrosion resistance in contact with Galinstan and three refrigerants at 150 °C, namely, *n-*pentane (R-601), 1,1,1,3,3-pentafluoropropane (R245fa), and isobutane (R600a), for exposure periods up to ∼20 days under pseudodynamic conditions with mechanical agitation to simulate gas–liquid-metal two-phase flow.^20^ Steel coupons were placed in sealed ampules containing the fluid mixtures and simultaneously exposed to the refrigerant gas phase, the Galinstan liquid phase, and the liquid–gas interface during testing.

Corrosion was observed only at the liquid–gas interface region of the SS316 coupons, as shown in Figure 1, whereas no measurable degradation occurred in the single-phase regions exposed to either the refrigerants or Galinstan. Nitrogen was used as a control gas. SEM examination and EDS mapping revealed varied levels of residual Galinstan or gallium and gallium oxide at the liquid–gas interface of the tested coupons exposed to refrigerant-containing environments, while the coupon exposed to nitrogen for 20.5 days showed no evidence of corrosion and negligible oxygen in the EDS spectra.

**Figure 1:**
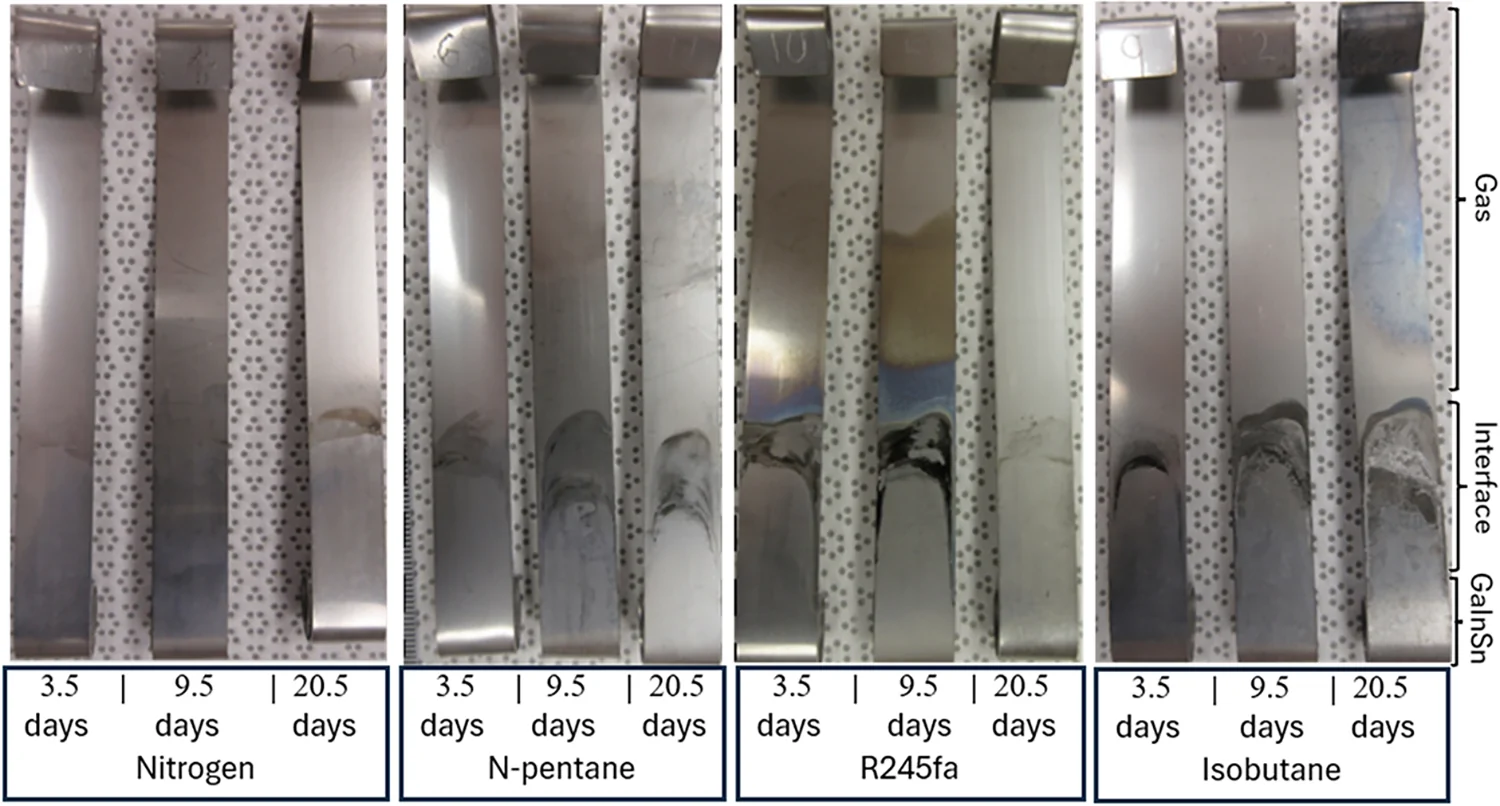
SS316 coupons after testing and final cleaning. Three exposure regions are visible: the top region exposed to the refrigerant gas phase, the bottom region exposed to the Galinstan liquid phase, and the liquid–gas interface region, where corrosion was observed for coupons exposed to refrigerant-containing environments.

These results indicated that SS316 may exhibit acceptable resistance under single-phase flow of Galinstan at 150 °C, but its performance is significantly reduced in gas–liquid-metal two-phase flow environments. Such behavior is particularly important for components such as gas-lift pumps, separators, and flow conduits used in liquid-metal–gas two-phase flow systems,^21^ where long-term reliability is essential.

The observed degradation of SS316 at the liquid–gas interface,^21^ together with the susceptibility of stainless steel to gallium and gallium alloy at elevated temperatures,^13–16^ highlights the challenges associated with Galinstan-based applications and underscores the need for more robust and cost-effective protective solutions.

Conventional corrosion protection strategies include metallic coatings, ceramic coatings, and diffusion-based surface treatments. More recently, advanced coating technologies, including polymer-based nanocomposites, graphene-reinforced coatings, self-healing coatings, and other multifunctional systems, have been developed to further enhance corrosion resistance.

Polymer-based nanocomposite coatings have demonstrated significant improvements in barrier properties, mechanical performance, and corrosion resistance.^22^ However, their long-term stability under aggressive environments, particularly at elevated temperatures and in multiphase systems, remains a challenge. For example, epoxy nanocomposite coatings reinforced with mixed metal nitrides and graphene oxide exhibit enhanced corrosion resistance and mechanical properties but are generally limited to moderate temperature applications.^23^ Similarly, multifunctional nanocomposite coatings that combine anticorrosion, mechanical, and flame-retardant properties have shown considerable promise for structural applications.^24^ Recent advances in graphene-based nanocomposites have further improved corrosion resistance through enhanced barrier effects and interfacial interactions.^25^ Despite these developments, most polymer-derived coating systems exhibit limited thermal stability and may undergo degradation in high-temperature or chemically aggressive environments,^26^ likely including liquid-metal systems such as those considered in the present study. Previous studies on different polymeric materials exposed to Galinstan have primarily been limited to low temperatures below 100 °C.^17^ As a result, the performance of polymer-derived coatings in Galinstan environments above 100 °C has not been systematically evaluated. Consequently, although advanced nanocomposite coatings represent promising corrosion protection technologies, their suitability for prolonged service in Galinstan-based liquid-metal and liquid-metal–gas two-phase environments, particularly at elevated temperatures exceeding 100 °C, remains highly uncertain.

Among the available coating approaches, metallic, ceramic, and surface alloyed types have been investigated for corrosion mitigation in different power generation applications.^27–33^ However, based on comprehensive analyses and extensive practical experience, most coatings exhibit one or more drawbacks, including inadequate corrosion resistance at high temperatures, structural defects (e.g., microcracks, pores, high surface roughness), poor adhesion, or incompatibility with complex geometries and long tubular components requiring protection of their inner surfaces. For example, hard nitride- and carbide-based ceramic coatings obtained by thermal spray and physical vapor deposition (PVD) technologies possess chemical and thermal stability and inertness, defined by their high thermodynamic properties.^34–36^ They may be recommended for molten metal applications, but these technologies are not well suited to apply the coatings onto large size tubing systems and complex shape components where inner surfaces need to be protected. In addition, some coatings involve exotic materials or costly, low-productivity processes, further restricting their industrial applicability. Identifying appropriate coatings and application methods capable of protecting low-alloy carbon steel components in liquid-metal or more demanding liquid-metal–gas environments remains a significant challenge.

Given the limitations of conventional coatings and advanced polymer-based nanocomposite coatings in liquid-metal or liquid-metal–gas environments, alternative surface treatment methods are of particular interest. For example, nanocrystalline diamond coatings have been evaluated in Ga–In–Sn liquid alloy and showed excellent resistance to liquid-metal penetration and corrosion.^37^ However, these investigations were conducted under static conditions at room temperature. Consequently, the reported results may not be directly transferable to the more demanding operating conditions encountered in practical heat-transfer systems. In addition, diamond coatings are generally associated with high processing costs and may present challenges when applied to large-scale tubular components or complex geometries requiring protection of internal surfaces.

Non-metallic refractory materials are considered among the most promising solutions for high-temperature molten metals and molten salts applications.^38^ In particular, hard borides, including transition metal (group IVa–VIa and others) borides and boride-based cermets, have demonstrated significantly enhanced corrosion resistance in contact with molten Al, Zn, transition metals, alloys, and solders compared to conventional metallic alloys. When boride-based cermets experienced corrosion, degradation generally occurred through selective attack on the metallic binder phases (e.g., Fe, Ni, Co) alongside intermetallide formation, while crystalline borides exhibited negligible interaction with molten metals.^39^ Notably, most metal borides exhibited poor wettability toward liquid metals,^40,41^ significantly suppressing interfacial interaction of these materials. Although bulk refractory boride-based materials possess attractive corrosion-resistant properties, they cannot be easily fabricated into large, complex shapes and long tubular components. Consequently, applying protective metal boride coatings on conventional steels and alloys could offer a more practical and cost-effective solution.

Low-alloy carbon steels are attractive engineering materials because of their low cost, widespread availability, and ease of fabrication; however, they generally lack sufficient corrosion resistance for aggressive environments. Among the available metal boride coating approaches, boronizing carbon steels is particularly attractive because it produces diffusion-bonded FeB and Fe_2_B layers characterized by high hardness, excellent adhesion to the substrate, and strong chemical and thermodynamic stability. This process is based on the principles of thermal diffusion chemical vapor deposition (CVD) but does not require hazardous gases, dedicated gas-control systems, or specialized reaction chambers, making it cost-effective and compatible with standard ceramic industry equipment. It also enables simultaneous processing of multiple components of various shapes and sizes, including long tubes requiring inner surface protection, which beneficially distinguishes it from many other coating technologies. These advanced coatings, featuring thicknesses of ∼50–300 μm, can be produced with high integrity, low porosity, and strong adhesion to steel substrates independent of steel type or composition.^42–49^ Boronized steel components with various sizes and shapes, including long tubulars and complex shape parts, have been successfully employed in downhole oil and gas production, mineral processing, and geothermal power generation.^44,46,50–53^ Previous studies have shown that iron boride coatings can provide resistance to a variety of aggressive environments, including high-temperature steam, CO_2_, H_2_S, hydrocarbons, brines, acidic and basic solutions, and mineral salts. In addition, owing to their high hardness and well-consolidated structure, boride coating also exhibits superior wear resistance under both dry and slurry abrasive or erosive conditions, enabling reliable performance in combined wear-corrosion conditions.^43,45,52–57^

The favorable performance of advanced iron boride coatings is commonly attributed to their high crystallinity, distinct crystal structure, well-consolidated microstructure, chemical stability, and high hardness. Managing the process parameters, different predetermined coating thicknesses (case depths) can be obtained. Recent studies have also demonstrated the promising performance of iron boride coatings immersed in a liquid lead-free solder alloy containing a sufficient Sn content at 320 °C.^58^ The double-layer FeB–Fe_2_B coating with a rather small thickness (∼30–40 μm) did not reveal surface dissolution and retained the coating thickness at the original level after prolonged exposure. In contrast, after 40 days, unprotected steels experienced intensive dissolution with the formation of Fe–Sn intermetallic phases, degrading the substrate material. Although these results were obtained in a different liquid-metal system, they suggest that iron boride coatings may offer resistance to Galinstan systems. Together with the demonstrated performance of boronized steels in various industrial corrosive environments, these findings provide motivation for evaluating boronized carbon steel as a potential protection strategy for components operating in liquid Galinstan–refrigerant systems.

To the best of the authors’ knowledge, no prior studies have reported the application or experimental evaluation of boride-based coatings in contact with Galinstan and selected refrigerants. In this work, iron boride coatings on low-alloy carbon steel were evaluated under corrosive conditions similar to those previously examined for SS316.^20^ The objective was to perform an initial assessment of the coating integrity and degradation behavior in these environments using weight change, cross-section optical microscopy, coating thickness measurements, microhardness determination and inductively coupled plasma optical emission spectroscopy (ICP-OES). The findings establish a baseline for further evaluation of boronized carbon steel in liquid Galinstan-refrigerant environments and provide a foundation for future validation under more representative operating conditions relevant to low-temperature liquid-metal heat-transfer and energy-conversion systems, such as liquid-metal-cooled nuclear reactor systems, liquid-metal magnetohydrodynamic (MHD) energy conversion units, and some other advanced energy technologies.

## 2 Methodology

### 2.1 Coupon Materials and Preparation

Low-alloy carbon steel (CS) grade A36/44W was selected as the coupon substrate material. The chemical composition of this low-cost steel is practically identical to that of commonly used industrial tubing steels such as J55, L80, and N80. Its composition, as reported in the supplier’s mill test report (MTR), is shown in Table 1. Each CS coupon, with approximate dimensions of 3 in (L) × 0.5 in (W) × 0.25 in (thickness) (76 mm × 12.7 mm × 6.3 mm), was cut from long flat bars supplied by the vendor. Two holes, each with a diameter of 0.25 in (6.3 mm), were drilled near the ends of each coupon. Each coupon was labeled on the side for identification. The surfaces were then slightly ground using a scotch pad to remove excessive roughness, followed by acetone cleaning to remove grinding residues and any remaining grease.

**Table 1:** Chemical Composition of Carbon Steel (CS), wt %

grade	Fe	C	Mn	Si	Cr	Ni	Mo	Cu	P + S
A36/44W	Bal.	0.16–0.18	0.60–0.65	0.19–0.22	0.09–0.11	0.11–0.15	0.024–0.03	0.30–0.33	0.026–0.033

The boronizing process developed by and proprietary to Endurance Technologies Inc. (ETI) is a thermal diffusion CVD-based process performed using a pack mixture. The boronizing mixture (pack) consists of a boron-rich active ingredient, a halide salt activator, and an inert filler. The CS coupons were fully immersed into the boronizing mixture inside a sealed retort. The retort was then heated in an electric kiln to a predetermined temperature ranging from 800–1100 °C for a specified duration (typically resulting in case depths from 50–250 μm). This heat treatment provided the necessary high temperature for chemical reactions within the powder mixture and facilitated the interaction of the resulting gaseous species with the steel substrate. The firing profile was selected to achieve a desired coating thickness (case depth) of approximately 150–200 μm for this study. Upon reaching the designated temperature and exposure duration, the kiln was cooled, the retort opened, and the coupons were removed from the spent pack. Residual powder was removed via brushing followed by acetone rinsing. Additional details regarding the boronizing mixture and the EndurAlloy process are documented elsewhere.^45,46,50–52^ While published literature and ETI patent disclosures establish these broad operational windows and fundamental thermochemical principles, specific parameters—including the exact heating rates and the proprietary weight proportions of the batch formulations—are omitted to preserve commercial trade secrets.

This boronizing process forms a double-layer coating on the entire surface of the CS coupon through several steps: (i) high-temperature reactions within the boronizing mixture generate boron-containing gaseous species; (ii) boron (B) atoms deposit on the steel surface and diffuse into the substrate; (iii) the diffusing boron reacts with iron (Fe), the primary constituent of steel; and (iv) iron borides form, grow and consolidate at high temperature according to diffusion-controlled kinetics. Crystalline iron borides (Fe_2_B and FeB) form according to their stoichiometry, with the Fe_2_B layer developing directly on the steel substrate and the FeB layer forming above the Fe_2_B layer,^44,45,50–53^ as illustrated schematically in Figure 2.

**Figure 2:**
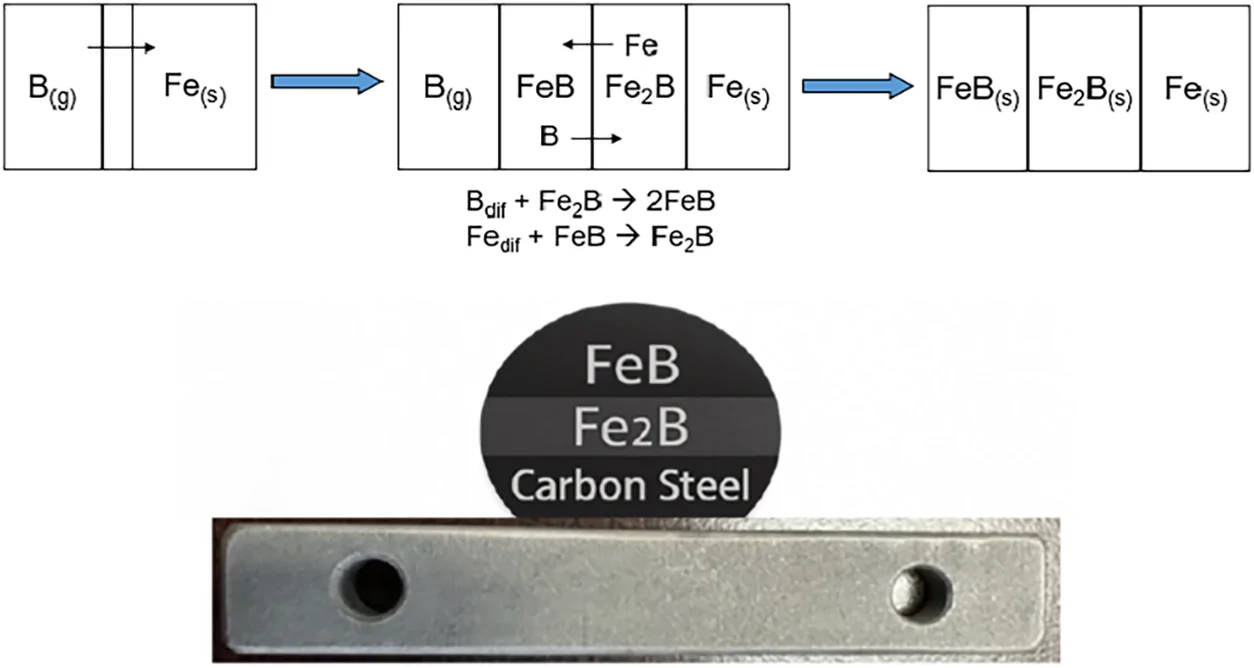
Iron boride coating formation on carbon steel via thermal diffusion process.

All coupons were processed simultaneously in the same retort, ensuring uniform coating properties and an approximately uniform coating thickness across the batch. The coated coupons were then weighed.

### 2.2 Corrosion Testing in Galinstan–Refrigerant Two-Phase Agitated Environment

The corrosion testing was conducted in liquid Galinstan with a composition (wt %) of Ga 67, In 20.5, and Sn 12.5. The liquid alloy (4N purity) was obtained from American Elements (USA), product code GA-INSN-04-LIQ; however, no preparation details were provided by the vendor.

Two organic refrigerants, *n*-pentane (R-601) and R1233zd­(E), were selected as representative working fluids from two important classes of low-boiling refrigerants: hydrocarbons (HCs) and hydrochlorofluoroolefins (HCFOs), respectively. They were supplied by Honeywell (USA). *n*-Pentane (C_5_H_12_) is a common nonpolar hydrocarbon with a low boiling point of 36 °C. It is frequently employed in organic Rankine cycles and other heat transfer applications due to its high latent heat and low viscosity. *n*-Pentane exhibits fair compatibility with SS316 under standard conditions.^59^ R1233zd­(E) (trans-1-chloro-3,3,3-trifluoropropene, CF_3_–CH = CHCl) is a hydrofluoroolefin refrigerant with a boiling point of 19 °C. It is nonflammable, providing a safer alternative to pentafluoropropane (R245fa), and was developed as a low-global-warming-potential (GWP ≈ 1) refrigerant for use in high-efficiency chillers and medium-temperature heat pump systems. According to the supplier’s data, R1233zd­(E) is not expected to cause corrosion under standard conditions.^60^ The purities of *n*-pentane and R1233zd­(E) exceeded 98% and 99.5%, respectively. Although the suppliers’ specifications do not list explicit impurities, trace amounts of moisture, noncondensable gases (*e.g.*, nitrogen, oxygen), or residual oils may still be present.

The ampule design used in the previous SS316 study^20^ was adapted for the current work and scaled up to accommodate the coupon, as shown in Figure 3. The Swagelok closures, cylindrical plugs, and pins were made of SS316. The test coupon was inserted into a cylindrical plug at the bottom of each ampule with a pin placed through the hole in the coupon. The plug outer diameter was closely matched to the inner diameter of the ampule tube. Once the bottom of the ampule was closed, the plug could be slid into place through the top opening. Each ampule contained one coupon.

**Figure 3:**
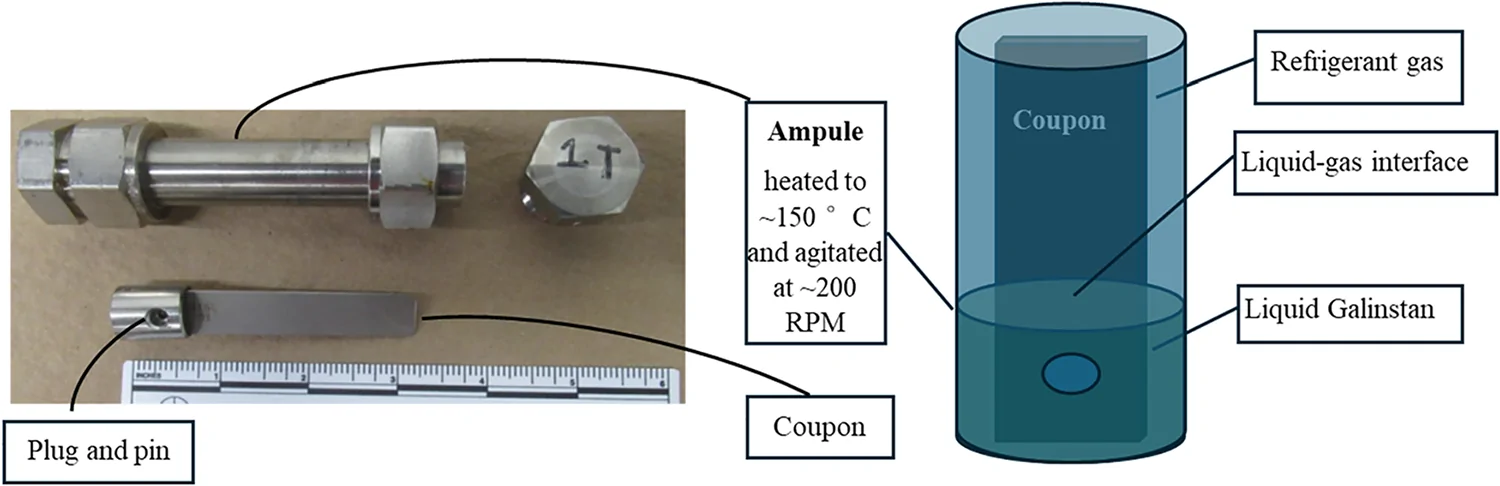
Schematic of an ampule containing gas and liquid metal, in which three exposure regions form on the coupon surface: a liquid-metal region, a gas-phase region and a liquid–gas interface region.

The test procedure began by placing the coupons, Galinstan, and unsealed ampules into the airlock of a glovebox, which was cycled with grade 5 nitrogen (N_2_ > 99.999%, O_2_ < 2 to 5 ppm, H_2_O < 0.5 ppm), to minimize oxygen and moisture contamination. Approximately 50 g of Galinstan was then added to each ampule by pouring, followed by insertion of a coupon. The ampules were then sealed before being removed from the glovebox and stored in a freezer.

Prior to refrigerant addition, the ampules were chilled in an isopropyl alcohol bath cooled with liquid nitrogen to reduce reaction rates between atmospheric oxygen and Galinstan and to maintain the refrigerants in the liquid state during transfer. *n*-Pentane, prechilled in the freezer, was added by pouring. The cylinder of R1233zd­(E) was first warmed using a space heater; the refrigerant then passed through a thin condenser coil partially immersed in an isopropyl alcohol–liquid nitrogen bath. The liquefied refrigerant was subsequently deposited into the chilled ampules. Filling operations were faster for liquid *n*-pentane than for cooled gaseous R1233zd­(E). The condenser and transfer line were purged with R1233zd­(E) prior to charging the ampules to minimize the risk of moisture entrapment.

After liquid refrigerant addition, the capped ampules were partially immersed in a 60 °C water bath for several minutes. The ampules were then vented by slightly loosening the seal until gas release was no longer audible, after which the seals were firmly tightened.

The ampules were then installed together in a fixture holder placed inside a laboratory electric kiln mounted on an orbital shaker, as shown in Figure 4. The ampule fixture holder contained 12 recesses, into which the ampules were inserted and secured using a rubber strip to evenly distribute the strapping force, followed by a metal retaining strap bolted into place. This ensured good thermal contact between the ampules and the aluminum fixture, which was equipped with a heating rod. Four thermocouples were used to monitor the temperature: two within the fixture block (one for control) and two on the outside of the ampules, insulated from the block thermocouples. The block thermocouple provided the most accurate indication of the ampule temperature.

**Figure 4:**
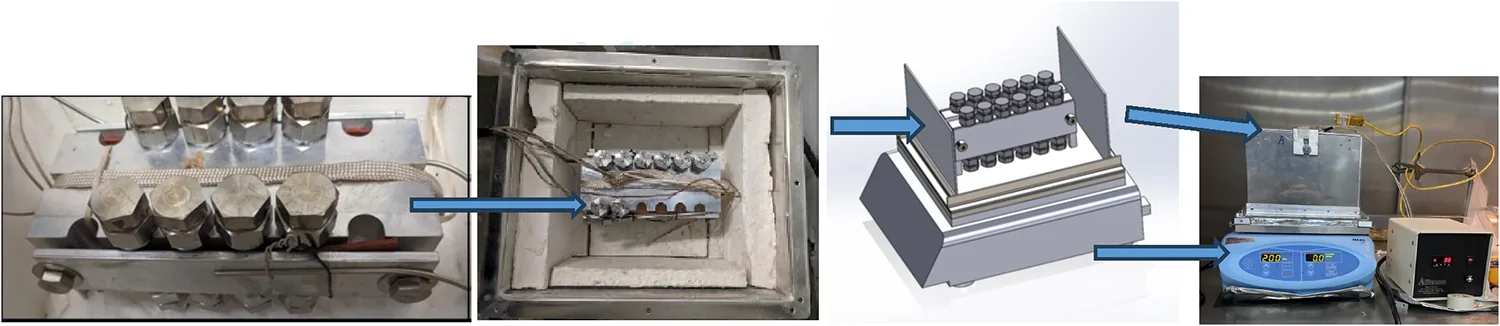
Testing setup showing the ampules, ampule fixture holder, electric kiln, and orbital shaker.

To simulate flow conditions typical of practical heat-transfer applications, a pseudoflow corrosion condition was established using an orbital shaker (VWR 3500) operated at approximately 200 rpm. The orbital shaker was intentionally employed to provide continuous agitation and movement of the corrosive media, promoting repeated interaction among the refrigerant, liquid Galinstan, and the liquid–gas interface. This was intended to create more aggressive corrosive conditions than those typically used in static laboratory testing. This configuration enabled the corrosion behavior in the gas phase, liquid-metal phase, and liquid–gas interface region to be clearly differentiated and systematically evaluated, whereas these location-specific effects may be difficult to isolate in a flowing liquid-metal–gas two-phase system.

The temperature was maintained at 150 °C throughout the testing period, except during coupon transfers. Test durations were set at 20, 40, and 80 days, four times longer than the corrosion testing of SS316 in the previous study,^20^ to evaluate whether boronized carbon steel performs comparably to or better than expensive SS316 under these conditions.

The selected laboratory test conditions (e.g., liquid Galinstan, gaseous refrigerants, temperature) simulate, to some extent, conditions related to certain processes in LMMHD energy conversion systems and small modular nuclear reactors. Although these conditions do not fully replicate the dynamic conditions involving liquid-metal–gas flows, the present test was designed as an initial screening to assess material compatibility under the combined exposure conditions. This approach follows previous testing in which SS316 was evaluated and found to be problematic.^20^

As summarized in Table 2, the coupon test matrix includes the refrigerant type, exposure duration, and the presence or absence of Galinstan. A control (“witness”) coupon was randomly selected from the same boronizing batch and was not subjected to corrosion testing, thereby preserving the original microstructure and composition.

**Table 2:** Coupon Test Matrix

test coupon #	test duration (days)	refrigerant	Galinstan presence
CS-B6	20	*n*-Pentane	yes
CS-B5	40	*n*-Pentane	yes
CS-B2	80	*n*-Pentane	yes
CS-B3	80	*n*-Pentane	yes
CS-B12	80	*n*-Pentane	no
CS-B8	20	R1233zd(E)	yes
CS-B9	40	R1233zd(E)	yes
CS-B4	80	R1233zd(E)	yes
CS-B11	80	R1233zd(E)	yes
CS-B15	80	R1233zd(E)	no
Control	N/A	N/A	N/A

Upon completion of the testing and opening of the ampules, the coupons were gently tapped on a hard surface to dislodge any large Galinstan globules. The coupons were then ultrasonically cleaned in distilled water containing a small amount of dish soap, followed by gentle wiping with a wet, lint-free wipe to remove any adherent residue.

### 2.3 Examination

Examinations included visual imaging, weight change measurements, and cross-sectional microstructural analysis for all coupons, including the control. The coupons were reweighed by using laboratory balances with an accuracy of 0.0001 g. Cross-sectional microscopy was performed using a light optical microscope (LOM) MEIJI Techno IM7200 (Japan).

Cross-sectional microstructural examination was performed to assess changes in the morphology of iron boride coatings on the carbon steel (CS) substrate. The coupons were sectioned, as illustrated in Figure 5, to enable examination of the coating area exposed to the liquid–gas interface, where corrosion was previously observed for SS316. After precision cutting and polishing to a 1 μm finish using Buehler equipment and diamond tooling, the samples were etched with a Nital solution (nitric acid in ethanol) to reveal microstructural features, following the established ETI procedure.

**Figure 5:**
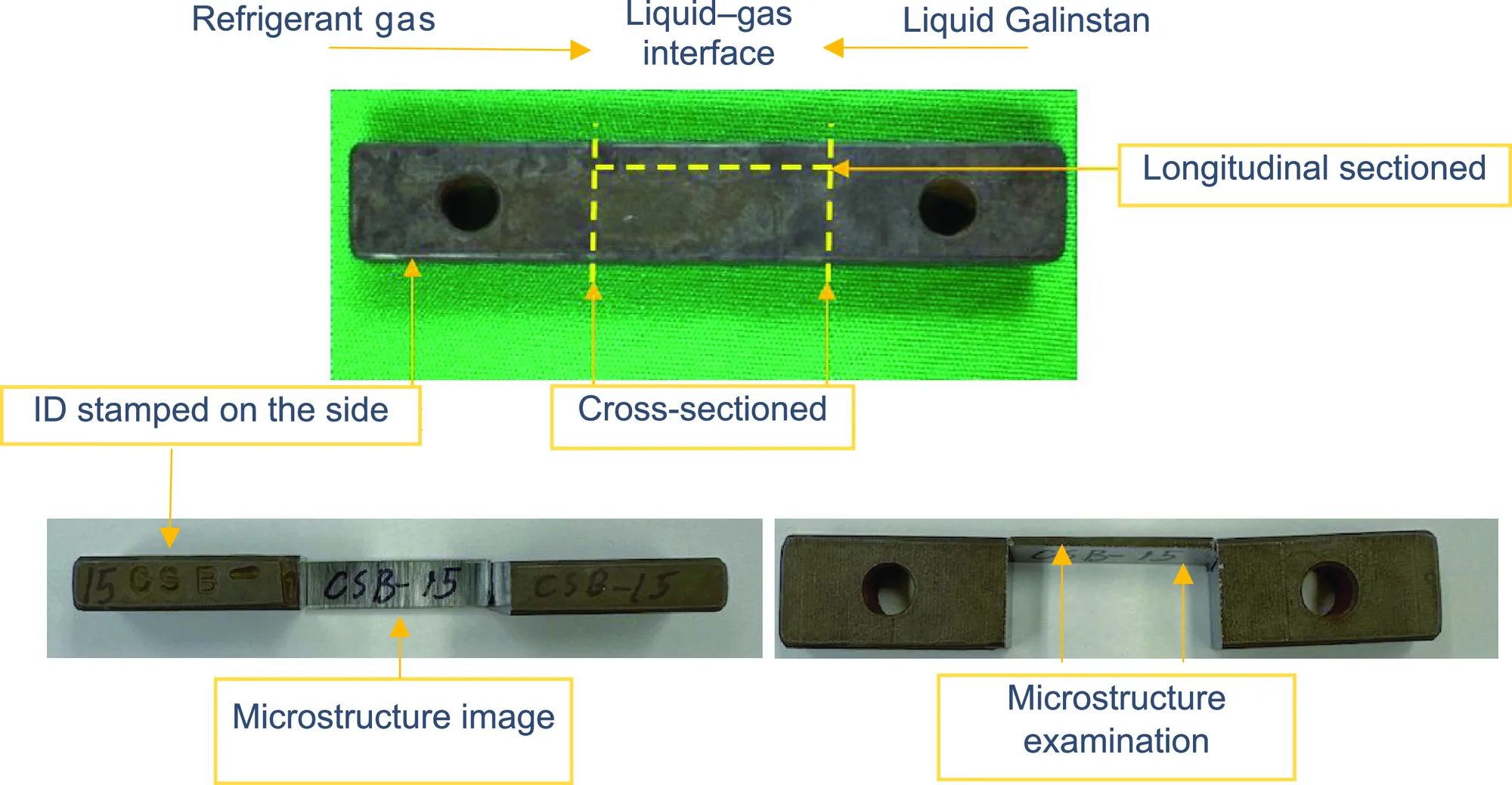
Coupon sectioning for cross-sectional microstructural analysis.

The control and test coupons were compared. This comparison is necessary because cross-sectional preparation for microstructural analysis is inherently destructive.

The Rockwell hardness test (HRB) was performed on the carbon steel substrate using a load of 100 kgf to evaluate hardness uniformity. The measurements were conducted in accordance with ASTM E18–22 using a Starrett analog Rockwell bench hardness tester (model 3814, USA). At least six indentations were made on each sample, and the reported values represent the average.

The Knoop microhardness test (HK 0.1) was performed for the iron boride coating according to ASTM E384–17 using a Clark microhardness tester CM400AT (USA). A Knoop (a rhomb shape) diamond indenter was applied to the polished cross section under a 100 g load (HK 0.1) for 10–15 s. The longer diagonal is aligned parallel to the coating surface. The Knoop hardness test method and the chosen indentation load (HK 0.1) were specifically selected as the most suitable method for hard coatings, minimizing the risk of chipping. The reported value represents the average of at least six indentations and measurements taken from each sample (each examined zone). Normally, the deviations in the HK 0.1 determination for boronized steels are below 5%.

Galinstan samples recovered after corrosion testing were analyzed by inductively coupled plasma optical emission spectroscopy (ICP-OES) to evaluate the possible dissolution of coating constituents into the liquid metal. Analyses were conducted by Kingston Process Metallurgy Inc. using an Agilent Technologies 5110 ICP OES on both exposed Galinstan samples and an unexposed control sample. Major alloy constituents (Ga, In, and Sn) and minor elements, including B, Fe, Cr, Cu, Mg, Mn, Mo, Ni, and P, were quantified. Since the boronized coating is composed primarily of FeB and Fe_2_B phases, changes in boron B and iron Fe concentrations in Galinstan were used as complementary indicators of possible coating degradation. The ICP-OES results were interpreted together with the coating thickness, microstructure, and hardness data to provide an overall assessment of coating performance after exposure. Whether additional surface characterization methods, such as scanning electron microscopy (SEM), energy-dispersive X-ray spectroscopy (EDX), and profilometry, are warranted depends on whether measurable degradation is detected by primary coating evaluation techniques. In the present study, cross-sectional microstructural examination, coating thickness measurements, microhardness testing, and ICP-OES analysis did not reveal measurable evidence of coating degradation, dissolution, substrate attack, or loss of coating integrity after exposure. Consequently, higher-resolution surface analyses were not included within the scope of this initial investigation.

## 3 Results and Discussion

### 3.1 Visual Examination

The coupons were visually inspected to assess the presence of surface defects, such as coating chipping, peeling, or nodule/bubble formation. Representative images are provided in Figure 6 for comparison. Two-stage imaging was conducted: (a) after boronizing, including before and after pretest washing (Figure 6a), and (b) after corrosion testing and ultrasonic cleaning (Figure 6b).

**Figure 6:**
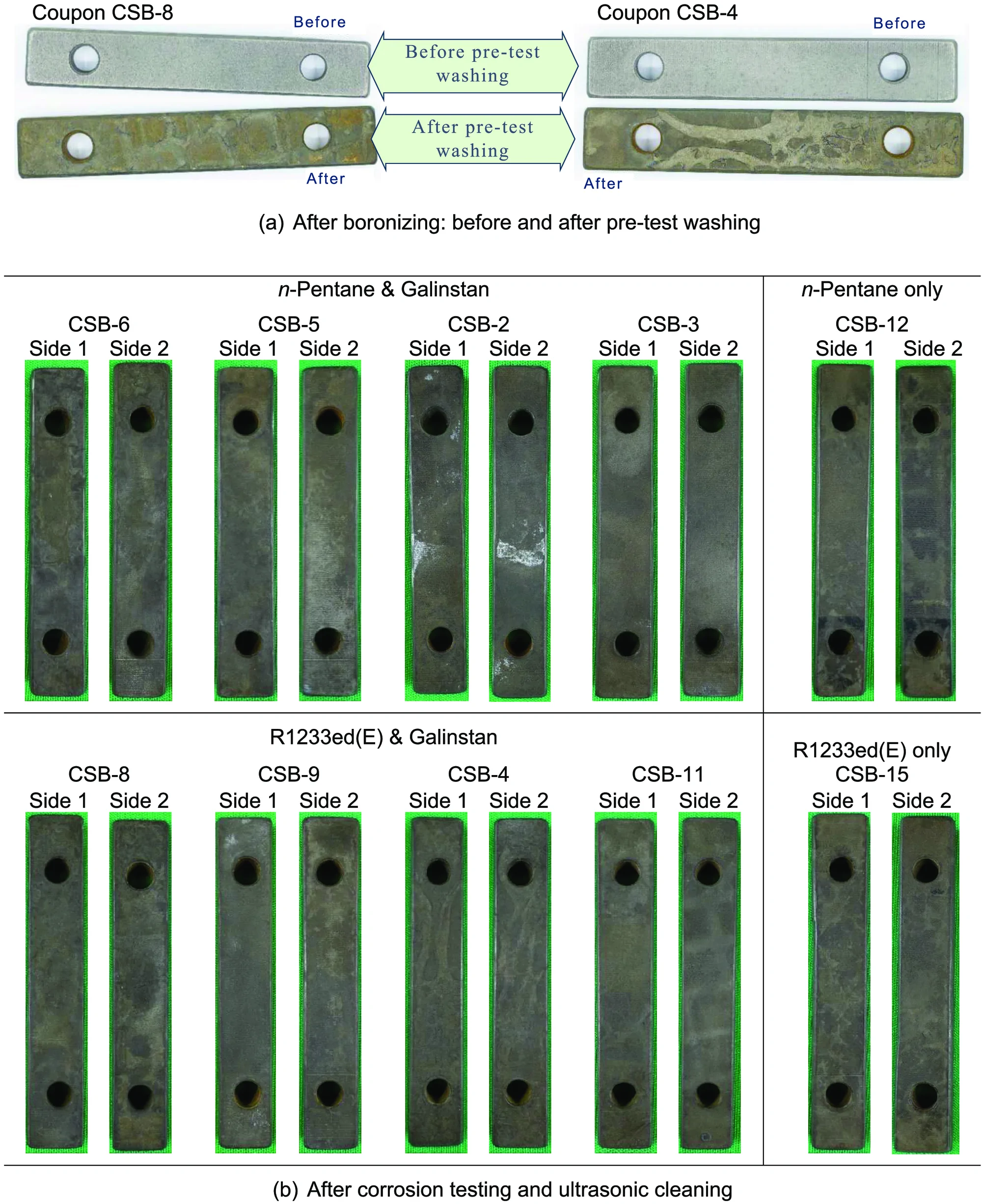
Images of the coupons.

After pretest washing using 80 °C water with cleaning agent LIQUINOX, the coupons exhibited discoloration or staining (Figure 6a), which is normal and only superficial for boronized steel. After corrosion testing and ultrasonic cleaning, all coupons appeared to become darker overall for both selected refrigerants. There was no obvious liquid–gas interface region or line appeared, which was obvious for SS316 during the previous test, and corrosions were identified in the interface region (Figure 1).

The observed discoloration is a commonly reported characteristic of boronized steels and has been observed in various industrial applications without evidence of associated material degradation. The surfaces of the tested boronized steel coupons exhibited higher roughness than the bare carbon steels, particularly when the steels are machined. This increased roughness is a feature of diffusion-grown FeB–Fe_2_B coatings. Although surface roughness (*R*_*a*_) was not quantitatively measured in the present study, the coupon surfaces were qualitatively examined by light optical microscopy before and after corrosion testing. No apparent changes in surface morphology were observed following exposure. In addition, residual Galinstan was readily removed from the boronized surfaces during the cleaning procedure despite their inherent roughness, and no visible accumulation of corrosion products, surface cracking, caverns, pinholes, scaling, or coating delamination was observed.

### 3.2 Weight Change

Coupon weights before and after corrosion testing, as well as the relative weight changes, are summarized in Table 3. The relative weight change was calculated as (Δ*m*/*m*_0_) × 100%, where Δ*m* is the weight difference of a coupon before and after corrosion testing and *m*_0_ is the coupon weight prior to testing. The weight changes were inconsistent: most coupons exhibited slight weight loss (<0.03%), while a few showed weight gain under similar test conditions.

**Table 3:** Weight Changes for the Tested Coupons

						relative weight change	
sample #	duration days	refrigerant	GaInSn presence	weight before test, (g)	weight after test, (g)	(g)	(%)
CS-B6	20	*n*-Pentane	yes	45.3274	45.3174	–0.01	–0.022
CS-B5	40	*n*-Pentane	yes	44.9088	44.9079	–0.0009	–0.002
CS-B2	80	*n*-Pentane	yes	44.6737	44.2718	–0.4019	–0.9
CS-B3	80	*n*-Pentane	yes	45.2935	45.3053	0.0118	0.026
CS-B12	80	*n*-Pentane	no	43.3945	43.3517	–0.0428	–0.099
CS-B8	20	R1233zd(E)	yes	45.0835	45.0751	–0.0084	–0.019
CS-B9	40	R1233zd(E)	yes	44.6897	44.6321	–0.0576	–0.129
CS-B4	80	R1233zd(E)	yes	45.6764	45.6665	–0.0099	–0.022
CS-B11	80	R1233zd(E)	yes	44.063	44.0929	0.0299	0.068
CS-B15	80	R1233zd(E)	no	44.0296	44.0204	–0.0092	–0.021

In corrosion testing, weight loss may result from material removal due to corrosion or from posttest handling and cleaning (e.g., accidental chipping of rough surfaces). Conversely, weight gain may arise from the formation of corrosion products or scale on the surface, or from residual deposits of solid media such as oxidized liquid metals or salts, particularly on rough surfaces.

Among the tested coupons, the highest relative weight loss was 0.9% for CS-B2 (80 days, *n*-pentane, and GaIn*S*n present), whereas the lowest was 0.002% for CS-B5 (40 days, *n*-pentane, and GaIn*S*n present). Several 80-day results were comparable to those obtained after 20 days of exposure, indicating no clear time-dependent trend. Likewise, no consistent influence of the refrigerant type was observed. The measured weight variations may therefore be associated with a combination of handling effects, cleaning procedures, residual Galinstan on the rough coating surface, measurement uncertainty, and/or localized material loss at accidental chipping.

For relatively rough coating surfaces such as iron boride, the weight change alone is not a reliable indicator of material integrity. A comprehensive assessment of corrosion performance requires additional microstructural and compositional analyses, which can more accurately reflect the material behavior under corrosive conditions. Therefore, coating stability was further evaluated using coating thickness measurements, microhardness testing, cross-sectional microstructural examination, and ICP-OES analysis of the exposed Galinstan, which are discussed in the following sections.

### 3.3 Cross-Sectional Microstructural Evaluation

Owing to the distinctive sawtooth morphology of iron boride coatings on the CS substrate, the coating thickness (case depth) was defined as one-third to one-half of the maximum peak height, providing a consistent and representative measure.

Material hardness is governed by composition, microstructure, and the presence or absence of structural defects (e.g., porosity, microcracks, or inclusions). Accordingly, hardness and microhardness testing are considered to be reliable methods for assessing coating integrity. The bulk hardness (HRB) of the CS substrate, and microhardness (HK 0.1) of both the iron boride coating and the CS substrate were determined using the methods described in Section 2.3.

Table 4 summarizes the coating thickness (case depth) and microhardness, and the substrate HRB hardness of the coupon area exposed to the liquid–gas interface. The microhardness values reported represent the average of at least six indentations performed in accordance with the applicable ASTM standard.

**Table 4:** Characteristics of the Coupon Area Exposed to the Liquid–Gas Interface: Coating Thickness (Case Depth) and Hardness Data^a^

					hardness		
cross section examination			case depth (μm) liquid–gas interface		iron-boride coating	CS substrate	
Coupon #	Test duration (days)	Refrigerant	Gas zone	Liquid zone	(HK 0.1)	(HK 0.1)	(HRB)
CS-B6	20	*n*-Pentane	198.12	190.5	1716	173	73
CS-B5	40	*n*-Pentane	187.96	195.58	1772	185	74
CS-B2	80	*n*-Pentane	195.58	187.96	1786	190	74
CS-B3	80	*n*-Pentane	182.88	185.42	1819	187	74
CS-B12	80, no GaInSn	*n*-Pentane	203.2	185.42	1812	180	74
*Average*		*n-Pentane*	*193.55*	*188.98*	*1781*	*183*	*73.8*
CS-B8	20	R1233zd(E)	182.88	198.12	1803	174	73
CS-B9	40	R1233zd(E)	180.34	185.42	1779	186	74
CS-B4	80	R1233zd(E)	200.66	190.5	1781	184	74
CS-B11	80	R1233zd(E)	193.04	198.12	1842	182	74
CS-B15	80, no GaInSn	R1233zd(E)	198.12	190.5	1820	183	74
*Average*		*R1233zd(E)*	*191.01*	*192.53*	*1805*	*182*	*73.8*
Control, top	N/A	N/A	185.44	185.44	1767	177	73
Control, bottom	N/A	N/A	200.66	200.66	1809	178	73
*Average*			*193.05*	*193.05*	*1788*	*177.5*	*73*

a Case depth values presented in this table represent the average thickness of the iron boride coating of at least six indentations performed from the gas zone to the liquid zone after corrosion testing; values for the unexposed control coupon are included for comparison.

Compared with the control coupon, no significant or systematic differences were observed in the characteristics of the exposed coupons, regardless of test duration or refrigerant type. Notably, even coupon CS-B2, which exhibited an isolated weight loss of 0.9%, retained a coating thickness and microhardness comparable to those of the control coupon. Overall, the coating thickness and microhardness showed no systematic decrease after exposure, remaining at their original levels within approximately 95–101% and 96–103% of the control values, respectively.

The statistical variation of the coupon-average coating hardness was 1781 ± 44 HK 0.1 for the *n*-pentane series and 1805 ± 29 HK 0.1 for the R1233zd­(E) series, compared with 1788 ± 30 HK 0.1 for the control condition. These variations are consistent with the heterogeneous FeB–Fe_2_B bilayer structure, where individual indentations may locally probe areas of different phase composition.

#### 3.3.1 Microstructure of the Control Coupon

The cross-sectional microstructure of the control coupon is shown in Figure 7, where two distinct layers can be observed: **FeB** (darker color) and **Fe**_**2**_ **B** (lighter color). The total coating thickness ranged from ∼180 to 200 μm, as reported in Table 4. The crystalline coating exhibits a distinctive “sawtooth” structure, typical of boronized low-alloy carbon steels, which provides strong adhesion and bonding to the steel substrate.^42–47,49–54^ Earlier conducted works, where the coating’s phase composition was determined using XRD, showed the presence of these FeB and Fe_2_B crystalline phases.^46,51,55,61^ No interior defects, such as voids, flaws, or delamination between the boride layers and the substrate, were observed. The Kirkendall porosity that may occur in some surface engineering processes at the coating–substrate interface was not detected, indicating the formation of strong bonding.

**Figure 7:**
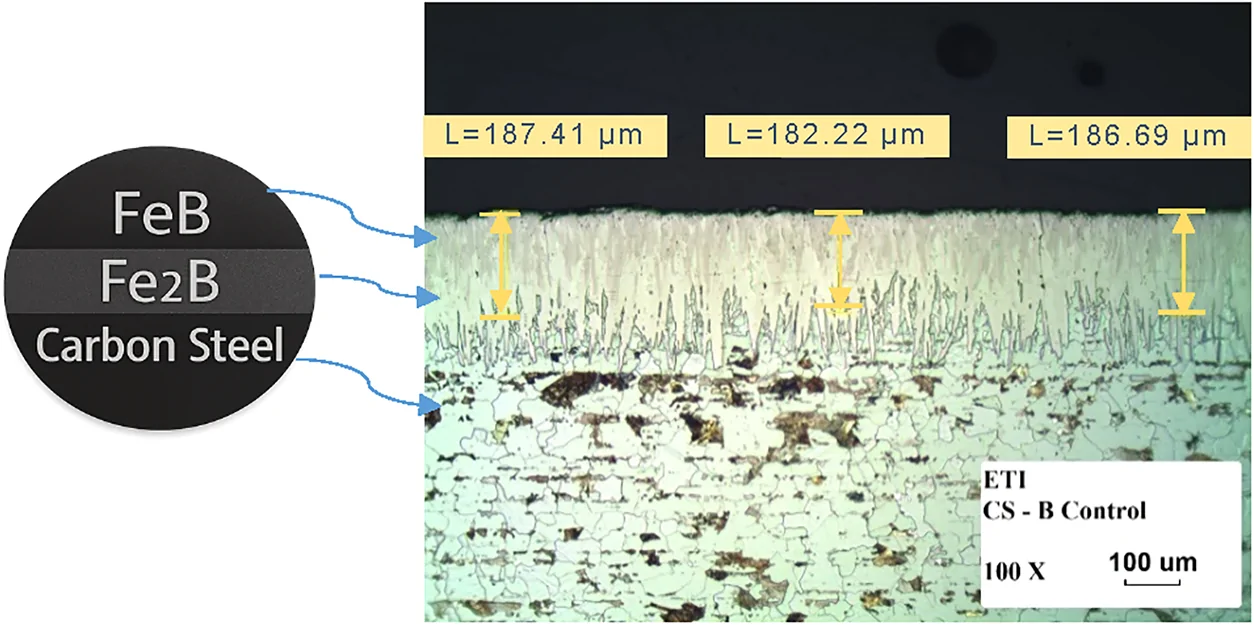
Cross-sectional microstructural image and coating thickness for control coupon top (100× magnification).

The coating on the control coupon exhibited a microhardness (HK 0.1) of 1600–1800 kgf/mm^2^, with the FeB layer (1700–1800 kgf/mm^2^) slightly harder than the Fe_2_B layer (1600–1650 kgf/mm^2^). These values are typical for boronized steels and are consistent with previous studies.^44,46,50–53,55,56^ The steel substrate exhibited a microhardness of 170–190 kgf/mm^2^, confirming that the iron boride coating is nearly an order of magnitude harder than the underlying carbon steel. The macrohardness of the carbon steel substrate was 73–74 HRB.

#### 3.3.2 Microstructure of the Tested Coupons

The cross-sectional microstructure images for 20, 40, and 80-day tested coupons are shown in Figure 8 for *n*-pentane and in Figure 9 for R1233zd­(E). The double-layer architecture of the coating remained intact, with no evidence of delamination. Notably, the total coating thickness remained at its original level of approximately 180–200 μm after 80 days of exposure. Minor variations in coating thickness among samples are attributed to differences in the samples’ positions within the production retort and in measurement location. No measurable corrosion products or surface scale were observed, and no surface or internal cracks, voids, or pinholes were detected. No evidence of coating degradation indicated that the coating integrity was maintained throughout the exposure period. No observable Kirkendall porosity, which can develop in some diffusion-produced coatings following prolonged exposure to high temperatures, was observed in the examined cross sections by optical microscopy at magnifications up to 500× (Figure 10). These observations suggest that the iron boride coating and its bond to the substrate remained stable under the test conditions. Because interfacial porosity may facilitate the accumulation of corrosive species and promote localized degradation, the observed lack of such features may be beneficial for maintaining coating integrity.

**Figure 8:**
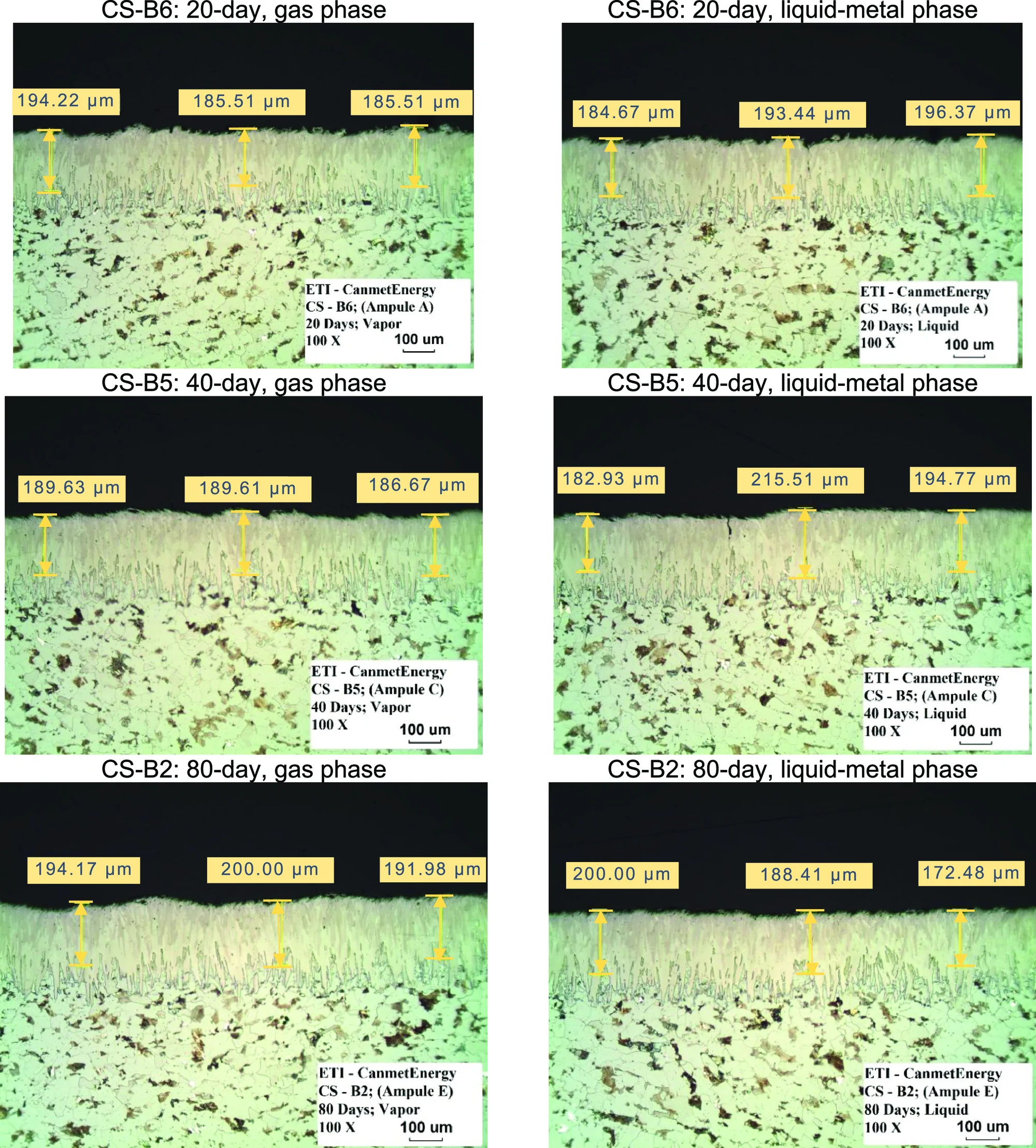
Cross-sectional microstructural images and coating thicknesses for coupons tested for 20, 40, and 80 days under *n*-pentane and Galinstan exposure (100× magnification).

**Figure 9:**
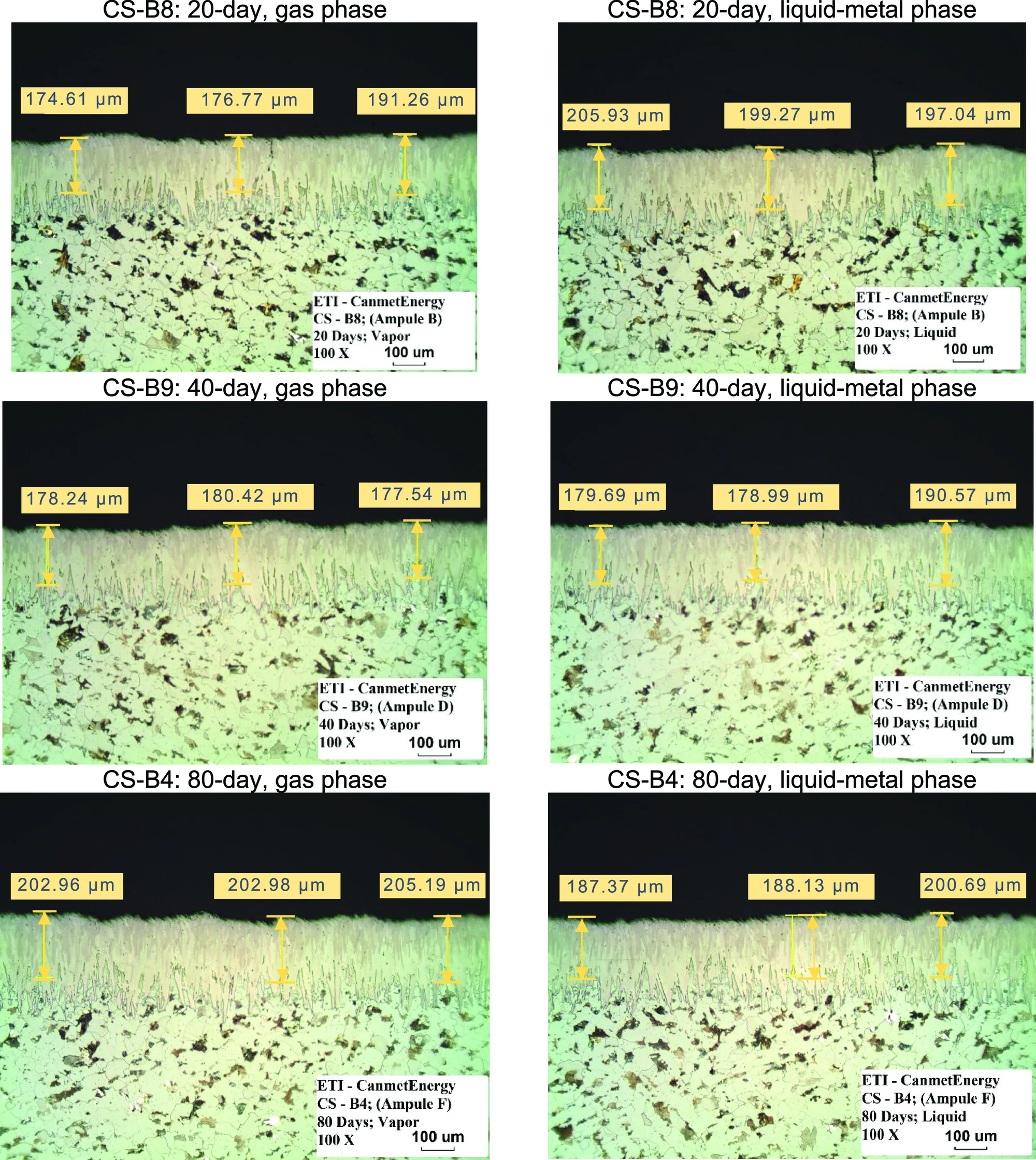
Cross-sectional microstructural images and coating thicknesses for coupons tested for 20, 40, and 80 days under R1233zd­(E) and Galinstan exposure (100× magnification).

**Figure 10:**
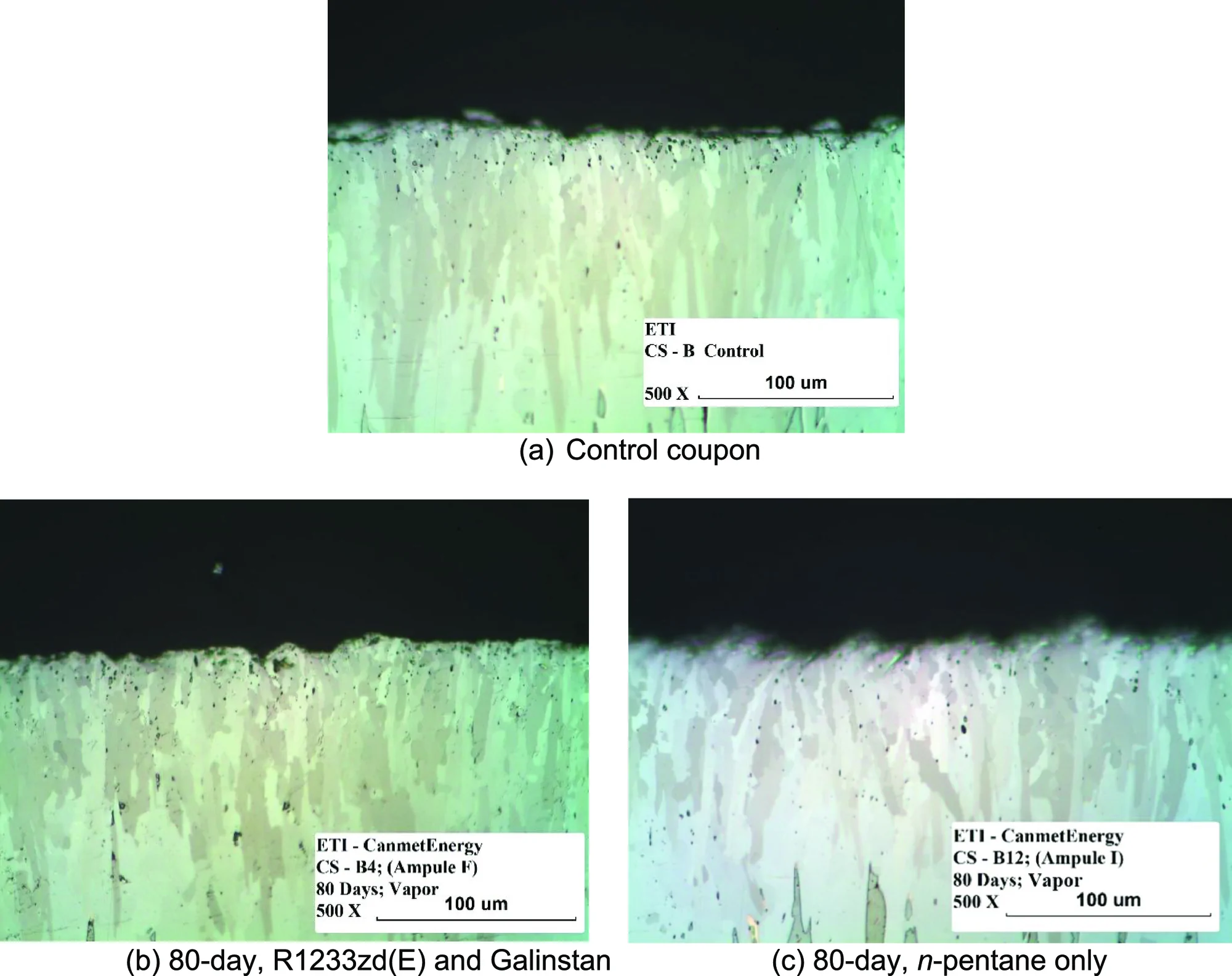
Coating surface images at 500× magnification: (a) control coupon; (b) coupon tested for 80 days in R1233zd­(E) and Galinstan; and (c) coupon tested for 80 days in *n*-pentane only.

The microhardness results reported in Table 4 were consistent with the optical microscopy observations and suggested that the iron boride coating maintained its integrity during the exposure period. The coating microhardness of the upper FeB-rich layer remained at its original level, ranging from 1750 to 1850 kgf/mm^2^ (HK 0.1), after the test, indicating stable mechanical properties. The deviations in the HK 0.1 data, which are in the common range for the iron boride coatings, are related to the point that a sharp diamond indenter, randomly applied on the coating cross section, in some cases may contact the harder FeB phase, while in some cases, it may contact the “softer” Fe_2_B phase. The hardness of the CS substrate also remained within its original range after exposure, indicating no measurable changes in substrate hardness. Together with the optical microscopy observations, these results suggest that, under the conditions investigated in this study, the iron boride coating maintained its protective function for the carbon steel (CS) substrate under the investigated conditions.

The coating surfaces of the coupons subjected to 80-day testing were further examined at higher magnification (500×) and compared with those of the control coupon. Both the control and tested coupons appeared visually similar, with no evidence of significant surface deterioration or formation of a visible corrosion-product layer on the tested coupons (Figure 10). This surface morphology is typical of coatings produced by the thermal diffusion process and largely reflects the original steel surface preparation. Importantly, no evidence of caverns, corrosion-induced microcracks, or chipping was observed. In the absence of observable corrosion products (like a corrosion scale) or other indications of coating degradation, additional surface analyses such as EDX and XRD were not pursued in this study. Furthermore, phase transformation of the coating was not anticipated under the present test conditions. Previous investigations of similar iron boride coatings in other corrosive environments at higher temperatures did not reveal any phase transformation according to the XRD analysis.^46,51,55,61^

### 3.4 ICP-OES Analysis of the Galinstan Samples

Since the protective coating consists primarily of FeB and Fe_2_B phases, boron and iron concentrations in the liquid metal were monitored in both unexposed (control) and exposed samples as complementary indicators of potential coating degradation or durability. Table 5 summarizes the ICP-OES results of boron and iron concentrations obtained for the Galinstan samples following exposure. The concentrations of other monitored elements (Cr, Cu, Mg, Mn, Mo, Ni, and P) were below the analytical detection limits for all samples (Cr < 0.001, Cu < 0.001, Mg < 0.005, Mn < 0.001, Mo < 0.001, Ni < 0.01, and *P* < 0.001).

**Table 5:** ICP-OES Analysis of Galinstan Samples on Boron and Iron Concentrations

sample ID	test condition	duration (days)	B (wt %)	Fe (wt %)
Control	Unexposed Galinstan	N/A	0.02	<0.005
CS-B6	*n*-Pentane + Galinstan	20	0.02	<0.005
CS-B5		40	0.01	<0.005
CS-B2		80	0.01	0.01
CS-B3		80	0.01	<0.005
CS-B8	R1233zd(E) + Galinstan	20	0.02	0.02
CS-B9		40	<0.01	0.03
CS-B4		80	<0.01	<0.005
CS-B11A		80	0.03	<0.005
CS-B11B		80	0.01	0.02

The measured boron concentrations in the exposed Galinstan ranged from 0.01 to 0.03 wt %, comparable with 0.02 wt % in the unexposed control sample. Iron concentrations in five of the nine exposed samples remained comparable to the control value (<0.005 wt %), whereas the remaining four samples exhibited slightly elevated iron concentrations ranging from 0.01 to 0.03 wt %. One possible contributor to the elevated iron concentrations is dissolution of the SS316 components used in the corrosion-test ampule assembly, which is consistent with the degradation previously observed for SS316 exposed to similar Galinstan–refrigerant environments. Notably, even coupon CS-B2, which exhibited an isolated weight loss of 0.9%, exhibited boron concentration within the range measured for the control sample. Overall, no systematic increase in either boron or iron concentration with exposure duration was observed.

The ICP-OES results should be interpreted together with the coating thickness, microhardness, and microstructural observations. The preservation of coating thickness and hardness, together with the absence of observable microstructural deterioration and the lack of progressive accumulation of boron in Galinstan, collectively suggest that significant degradation or dissolution of the iron boride coating did not occur under the investigated conditions.

### 3.5 Discussion on the Resistance of the Iron Boride Coating

While the SS316 steel experienced noticeable corrosion issues under similar test conditions over a much shorter duration of 20 days,^20^ the boronized carbon steel exhibited no measurable degradation after exposure of up to 80 days, according to microstructural and ICP-OES examination. Notably, even coupon CS-B2, which exhibited an isolated weight loss of 0.9%, retained a coating thickness and microhardness comparable to those of the control coupon and exhibited a boron concentration in the exposed Galinstan within the range measured for the control sample. The observed stability may result from a combination of favorable materials chemistry (thermodynamic stability, strong covalent bonding, and reported chemical stability of iron borides), robust coating architecture (FeB-Fe_2_B bilayer structure with strong diffusion-induced metallurgical bonding), and the relatively large coating thickness, which together may limit coating degradation under the investigated conditions. However, the proposed corrosion resistance mechanisms should be viewed as plausible interpretations consistent with the observed experimental results rather than as experimentally confirmed mechanisms.

The crystalline iron borides FeB and Fe_2_B both possess high lattice energies. The standard enthalpy (−ΔH) values of Fe_2_B and FeB are approximately 67–71 kJ mol^–1^ and 71–82 kJ mol^–1^, respectively,^61–64^ reflecting their thermodynamic stability. In addition, both phases exhibit high valence-electron densities and short, highly covalent Fe–B and B–B bonds,^57,65,66^ which may contribute to resistance against bond disruption during chemical interactions at elevated temperatures in corrosive environments.

Comprehensive studies have shown that materials combining high lattice enthalpy, high valence-electron density, and strong covalent bonding within their crystalline lattices often exhibit exceptional durability and chemical stability.^66–69^ For example, boride-based cermet coatings obtained from the metal boride – metallic alloy mixtures MoB/CoCr, MoB/NiCr, and TiB/CoCr by the HVOF spraying process were tested in molten zinc at 460 °C for 360 h. Although these coatings may have practical limitations, including high cost and difficulty in protecting the inner surface of long tubular components, they demonstrated encouraging corrosion resistance, which the authors attributed to the presence of thermodynamically stable metal-boride crystalline phases such as TiB_2_, CoMoB, CoMoB_2_, and NiMo_2_B_2_ within the metallic matrix.^70^ Similar conclusions were reported by Z. Salvi et al.,^58^ who conducted thermodynamic calculations of possible reactions between FeB and Sn and found a positive Gibbs free energy (Δ*G*) value, indicating that such reactions are thermodynamically unfavorable. Iron borides, which possess many of these characteristics, are therefore considered among this group of materials. The absence of free metallic phases or binder constituents within the iron boride layer, together with its well-organized crystalline structure, may reduce the number of microstructural features susceptible to selective attack. These characteristics may also minimize interactions between the coating constituents and reactive, low-viscosity liquid metals, thereby reducing the potential for the formation of new intermetallic compounds commonly observed in steels, alloys, and cermets.

The reported lower wettability of some metal borides by liquid metals^40,41^ may suggest that iron borides exhibit similar behavior in Galinstan environments; however, this has not been experimentally verified and therefore remains to be confirmed through a dedicated study. Furthermore, the principal elements of Galinstan (Ga, In, and Sn) have comparatively large atomic radii, which may limit their ability to diffuse into the dense crystalline lattice of iron borides or disrupt the strong Fe–B bonds.

Beyond the intrinsic properties of iron borides, the microstructural architecture of the coating may also contribute to its observed stability. Corrosion in solid, well-consolidated, chemically stable compounds typically initiates at surface defects or microcracks and progresses toward the interior of the material.^30,31,46,50,51,57^ In coatings composed of multiple protective layers, microcrack propagation is significantly delayed because cracks tend to propagate at various angles with crack bridging or deflection rather than directly toward the substrate.^33,46,70^ Such behavior can increase the resistance of the coating system to crack growth and may slow the progression of corrosion-related degradation.

The FeB–Fe_2_B coating possesses a bilayer structure; therefore, similar crack-deflection mechanisms may contribute to its observed stability. In addition, the FeB and Fe_2_B layers are strongly bonded to one another and to the steel substrate. The distinctive “sawtooth” morphology promotes mechanical interlocking and enhances interfacial adhesion, which may help delay microcrack initiation and propagation under corrosive attack.^46,50–53^ Supporting evidence for the beneficial role of the FeB-Fe_2_B architecture has also been reported in other liquid-metal systems. For example, carbon steel was boronized and evaluated in molten aluminum (630 °C) and molten zinc (500 °C) baths.^71^ The resulting ∼100 μm thick, dual-phase FeB/Fe_2_B protective coating featuring a sawtooth-shaped interface was substantially less susceptible to dissolution than the untreated carbon steel. The ICP-OES observations from the current study revealed only low concentrations of B and Fe in the exposed Galinstan, with no systematic increase with exposure duration, suggesting that dissolution of the iron boride coating, if present, was limited under the investigated conditions. This observation is consistent with the preservation of coating thickness, microhardness, and microstructural integrity after up to 80 days of exposure. Although the environments differed significantly from molten aluminum and zinc systems, the favorable performance reported in both studies suggests that the combination of thermodynamically stable iron boride phases and FeB-Fe_2_B bilayer structure may contribute to resistance against degradation in liquid-metal environments. In addition, the diffusion-induced “metallurgical” bonding between the chemically stable layers and the steel substrate, together with the absence of observable Kirkendall porosity, may further contribute to the structure stability of the coating and its resistance to degradation.^72^

Microstructural analysis of the iron boride coatings in the study revealed a well-consolidated bilayer coating with a total thickness of approximately 180–200 μm, which remained essentially unchanged after 20, 40, and 80 days of exposure. Minimal surface and internal defects were observed, suggesting a low density of observable defects, which may have limited preferential pathways for liquid-metal penetration and localized degradation. In addition, the relatively large coating thickness (180–200 μm) may also have contributed to the observed stability by increasing the diffusion path length and reducing the likelihood of substrate exposure during the investigated exposure period.

Importantly, the coating thickness, microhardness, microstructural observations, and ICP-OES results collectively indicate consistent coating behavior throughout the exposure period despite the differing test environments and durations.

Although oxygen exposure was minimized throughout the corrosion testing, trace amounts of oxygen or air may have induced slight oxidation of the boronized surface at 150 °C, leading to the formation of a thin glassy (Fe,B)*_x_* O_γ_ surface film. Based on our previous investigations,^46,50,51,53^ the discoloration observed after exposure test (dark hue in Figure 6 (b)) may be associated with the formation of (Fe,B)*_x_* O_γ_, an extremely thin submicron oxide nanofilm on the outermost surface, analogous to the passive chromium oxide film formed on stainless steels. SEM-EDS and XRD analyses conducted in previous studies confirmed the presence of oxygen on the surface while showing no detectable changes in the iron boride coating structure. This glassy film was found to adhere strongly to the FeB surface, with no evidence of peeling or spallation, and may contribute to surface healing and provide additional protection against further oxidation or corrosion. The formation of the amorphous (Fe,B)*_x_* O_γ_ surface film and its positive influence were also observed when iron boride coatings were exposed to other corrosive environments at elevated temperatures.^46,50,51,53^

The observed stability of the FeB–Fe_2_B coating after up to 80 days of exposure suggests that diffusion-grown boride coatings may be promising candidates for liquid-metal environments. In contrast to many polymer-derived barrier coatings, which commonly experience thermal or chemical degradation at elevated temperatures,^73–75^ the observed stability of iron boride coatings may be associated with their reported intrinsic chemical stability, well-consolidated double-layer crystalline structure, substantial coating thickness, and metallurgical bonding to the substrate. The absence of measurable degradation after 80 days under pseudodynamic conditions suggests that boronized steels warrant further evaluation under more demanding operating conditions.

## 4 Conclusions

To the best of the authors’ knowledge, this study presents the first evaluation of advanced iron boride coatings on carbon steel in corrosive environments containing liquid Galinstan and selected refrigerants (*n*-pentane or R1233zd­(E)) at 150 °C for exposure durations of up to 80 days under pseudodynamic conditions. Cross-sectional microstructural examination, coating thickness measurements, microhardness testing, and ICP-OES analysis consistently indicated preservation of the FeB–Fe_2_B coating. Coating thickness and hardness remained comparable to those of the control coupon. ICP-OES analysis also revealed only low concentrations of boron and iron in the exposed Galinstan. These concentrations remained within the range measured for the control sample and showed no systematic increase as a function of exposure duration, suggesting that dissolution of the iron boride coating, if present, was limited under the investigated conditions. No measurable cracking, pitting, or corrosion product deposition was observed by the employed characterization techniques. Collectively, these observations indicate that no significant degradation or dissolution of the iron boride coating was detected under the investigated conditions.

The favorable performance observed in this study may be associated with the reported intrinsically high hardness and chemical stability of iron borides, together with the well-consolidated FeB-Fe_2_B bilayer structure and diffusion-induced bonding to the substrate. The substantial coating thickness and diffusion-induced “metallurgical” bonding between the boride layers and the steel substrate may have also contributed to the coating stability observed under exposure to liquid Galinstan, selected refrigerants, and their combination.

Compared with previously reported studies in which SS316 exhibited measurable degradation under similar exposure conditions, these findings warrant further evaluation of iron boride coatings for protecting low-cost steel components, particularly tubular geometries, in liquid metal heat transfer applications. However, as some of these systems may operate at temperatures exceeding those investigated in the present study, further evaluation of long-term stability and higher-temperature performance within the operational temperature limits of the iron boride coating is required.

Under the pseudodynamic conditions of the present study, no clear trend in weight change was observed. Corrosion behavior may differ under true dynamic conditions, in which the corrosive medium is actively transported through the system, and the associated flow velocity may significantly accelerate corrosion and material degradation. Consequently, in future studies, the evaluation of iron boride coatings under dynamic gas–liquid-metal flow in piping systems would provide more practical and actionable insights for industrial applications.

The present findings should be interpreted within the scope of the investigated conditions. Only two refrigerants and a limited number of specimens were evaluated under pseudodynamic conditions at 150 °C. Although the results remained consistent throughout exposure for up to 80 days, additional studies involving larger sample populations, higher temperatures, longer exposure durations, additional refrigerants, and true dynamic flow conditions in iron boride-coated tubular systems are required to assess the broader applicability of boronized carbon steel in future work. Overall, this study establishes a baseline for evaluating boronized carbon steel in liquid Galinstan-refrigerant environments and provides a foundation for future validation under industrially relevant operating conditions. The consistency among the microstructural observations, coating thickness measurements, microhardness results, and ICP-OES analysis provides a coherent set of evidence, suggesting that the FeB-Fe_2_B coating maintained its integrity under the investigated conditions.

## Abbreviations

GaInSn: Galinstan
LMMHD: liquid-metal magnetohydrodynamic
CS: carbon steel
CVD: chemical vapor deposition
ICP-OES: inductively coupled plasma optical emission spectroscopy
EDX: energy-dispersive X-ray spectroscopy
XRD: X-ray diffraction
